# An automated microfluidic platform for *C. elegans* embryo arraying, phenotyping, and long-term live imaging

**DOI:** 10.1038/srep10192

**Published:** 2015-05-07

**Authors:** Matteo Cornaglia, Laurent Mouchiroud, Alexis Marette, Shreya Narasimhan, Thomas Lehnert, Virginija Jovaisaite, Johan Auwerx, Martin A. M. Gijs

**Affiliations:** 1 Laboratory of Microsystems, Ecole Polytechnique Fédérale de Lausanne, CH-1015 Lausanne, Switzerland; 2 Laboratory for Integrative and Systems Physiology, Ecole Polytechnique Fédérale de Lausanne, CH-1015 Lausanne, Switzerland

## Abstract

Studies of the real-time dynamics of embryonic development require a gentle embryo handling method, the possibility of long-term live imaging during the complete embryogenesis, as well as of parallelization providing a population’s statistics, while keeping single embryo resolution. We describe an automated approach that fully accomplishes these requirements for embryos of *Caenorhabditis elegans*, one of the most employed model organisms in biomedical research. We developed a microfluidic platform which makes use of pure passive hydrodynamics to run on-chip worm cultures, from which we obtain synchronized embryo populations, and to immobilize these embryos in incubator microarrays for long-term high-resolution optical imaging. We successfully employ our platform to investigate morphogenesis and mitochondrial biogenesis during the full embryonic development and elucidate the role of the mitochondrial unfolded protein response (UPR^mt^) within *C. elegans* embryogenesis. Our method can be generally used for protein expression and developmental studies at the embryonic level, but can also provide clues to understand the aging process and age-related diseases in particular.

Exposure to particular environmental conditions during early life is often decisive for later successful development of a living organism. In *C. elegans*, events occurring during larval stages are known to have a strong impact on the animal’s lifespan^1^. Whether conditions in the embryonic phase of life have an influence on the later development is a much more challenging question to answer, mainly because systematic *C. elegans* embryonic morphogenesis studies are still difficult from a technical point of view. In fact, *C. elegans* worm culture is performed on the surface of nematode growth medium agar plates covered with *Escherichia coli* bacteria for feeding. This technique proves especially tedious if large numbers of animals are to be analyzed. Moreover, for embryonic studies, currently available protocols are based on animal dissection and embryo mounting on agar pads^2^, which typically require specialized and advanced skills and lacks reproducibility and high-throughput potential. Recently, it has been shown that various aspects of functional exploration of *C. elegans* can be significantly improved using microfluidics^3^,^4^,^5^,^6^,^7^,^8^. “Worm-chips” have successfully demonstrated their high potential at enhancing worms’ handling and accurate imaging, for applications in lifespan studies^9^, phenotyping and screening^10^,^11^, nerve regeneration analyses^12^, as well as for the investigation of worms’ behavioral dynamics^13^. So far, however, such robust methods to study *C. elegans* embryos do not exist yet, whereas a microfluidic solution has been only proposed to study early embryo development for larger size model organisms, like *Drosophila melanogaster* and *Danio rerio*^14^,^15^. *C. elegans* embryos are 10–100 times smaller than those of the other small model organisms and almost impossible to handle manually.

To enable systematic analysis of *C. elegans* embryonic morphogenesis, we developed a microfluidic platform for automated on-chip worm culture, creation of synchronized embryo arrays, and for long-term parallel live imaging at the single embryo level. We successfully employed our platform to investigate mitochondrial biogenesis during the embryonic development. Using our method to study a large number of embryos of different wild-type and mutant worm strains, we elucidated an outstanding issue regarding the role of UPR^mt^ during early worm embryogenesis.

## Results

### Platform design and automated operation

**Figure 1:**
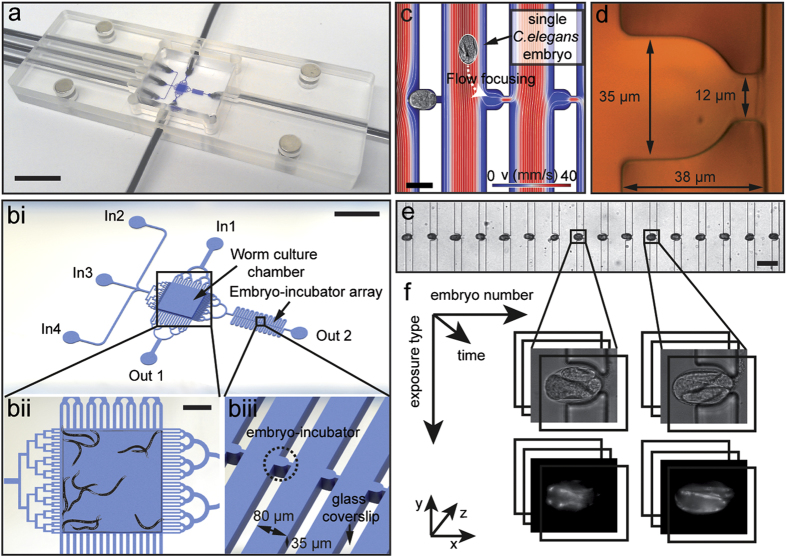
Overview of the microfluidic device. (**a**) Picture of the microfluidic device, sizing 25 mm × 75 mm (standard microscope slide size), including lateral microfluidic connections, which make the device compatible for imaging with every upright or inverted microscope. Scale bar = 10 mm (**b**i) Schematic representation of the central part of the microfluidic chip, having as main constitutive parts: the worm culture chamber, the embryo-incubator array, four inlets (In1 to In4) and two outlets (Out1 and Out2). Scale bar = 2 mm (**b**ii) Zoom on the worm culture chamber, including a drawing of young adult *C. elegans* for size comparison. The chamber is delimited by specific microfluidic channel arrangements, tailored for different functions: (top) worm injection, (bottom) worm synchronization, (left) *E. coli*/drug delivery and (right) egg transfer. Scale bar = 500 μm (biii) Three-dimensional schematic zoom on a portion of the embryo incubator array. (**c**) Finite element method simulation (Comsol Multiphysics) of the fluid dynamics in the incubator array region, showing the principle of passive hydrodynamic arraying of single embryos. Fluidic velocity and streamlines are calculated for a flow rate of 100 nL/s at the inlet In3. Scale bar = 50 μm (**d**) Micrograph of a single incubator on the SU-8/silicon master mold used for PDMS casting. (**e**) Micrograph of a section of the array with immobilized embryos. Scale bar = 100 μm (**f**) Illustration of the multi-dimensional imaging that is enabled on the array of embryos and spans six dimensions: the 3 spatial coordinates, time, exposure type and embryo number in the array.

The robustness and automation of our system completely relies on passive hydrodynamics, with no need of any active component on-chip, such as integrated valves. This approach allows simplifying fluidic protocols and significantly minimizing fabrication constrains of the device, which simply consists of a monolithic polydimethylsiloxane (PDMS) microfluidic chip, sealed to a ~150 μm-thick glass coverslip. Our microfluidic chip features two main components: a “worm culture chamber” and an “embryo-incubator array” (Fig. 1a,bi). External flow control through four independent inlets is achieved via computer-controlled syringe pumps, while two external valves are used to open and close two separate outlets. The worm culture chamber is delimited by specific microfluidic channel arrangements for generating uniform flow distributions in the chamber and for filtering entities of different size (Fig. 1bii and Fig. SI1): a “worm injection filter”, for gentle insertion of mixed worm suspensions into the chamber; a “worm synchronization filter”, to select the age of the worm population to be tested, by only retaining either adult worms or L4 larvae inside the chamber; an “*E.coli*/drug delivery filter”, to homogeneously introduce feeding and treatment solutions inside the chamber; an “embryo transfer filter”, to reliably displace embryos from the chamber to the embryo-incubator array upon egg laying. The embryo-incubator array consists of a serpentine channel in which each pair of branches is connected by isolated micro-compartments, specifically tailored for the trapping of *C. elegans* embryos and their high-resolution imaging through the glass coverslip (Fig. 1biii). Embryos that are transferred to the embryo-incubator array are automatically positioned in the micro-incubators by passive hydrodynamic trapping (Fig. 1c). The design of this section of the chip is optimized according to both general microfluidic rules and specific needs related to the characteristics of *C. elegans* embryos (Supplementary Note 2). Overall, our fluidic design results in enhanced efficiency of capture and stable positioning of single embryos, with unprecedented performance in terms of control and reliability of the trapping mechanism for non-spherical objects (Supplementary Video 1). The flow rate distribution inside the array has to ensure the capture of a single embryo for each micro-incubator. Since the number of available embryos is being limited by the egg production inside the chamber, a perfect efficiency of the hydrodynamic trapping method has to be established in order to recover all eggs. At the same time, however, high trapping efficiency is typically associated to higher fluidic pressures through the micro-incubators. Therefore, forces exerted on the incubated eggs have to be considered as well, to prevent the flow from introducing undesired mechanical stresses on the captured embryos. A crucial role for the system performance is clearly played by the geometry of the micro-incubators, hence different types of micro-incubators have been fabricated and tested (Supplementary Note 3). For high-resolution parallel time-lapse imaging of the whole embryo population and automated image processing, all the embryos have to be perfectly arrayed in stable positions and kept correctly aligned and well-oriented for several hours. We optimized the incubator size and shape mainly according to these needs, with our final design featuring elongated semicircular incubators, which are 35 μm wide, 38 μm long and 35 μm high (Fig. 1d). Using these dimensions, single embryos can be reliably positioned and aligned inside the incubator array (Fig. 1e). Only slight variations in their angular positions are observed, given the natural size variability of the embryos (Supplementary Note 4). The whole incubator array features 20 micro-incubators, which are progressively filled by embryos as soon as they are naturally laid. This number is chosen to provide a significant data statistics for each experiment, while still maintaining a good level of age-synchronization among all the embryos in the array. For a worm culture which is at the peak of its egg production, complete filling of the incubator array typically takes around 1 hour. The whole embryo population is studied using fully automated multi-dimensional imaging, covering six independent dimensions: the 3 spatial coordinates, the development time, the exposure (brightfield, fluorescent) duration, and the embryo number in the array (Fig. 1f).

**Figure 2:**
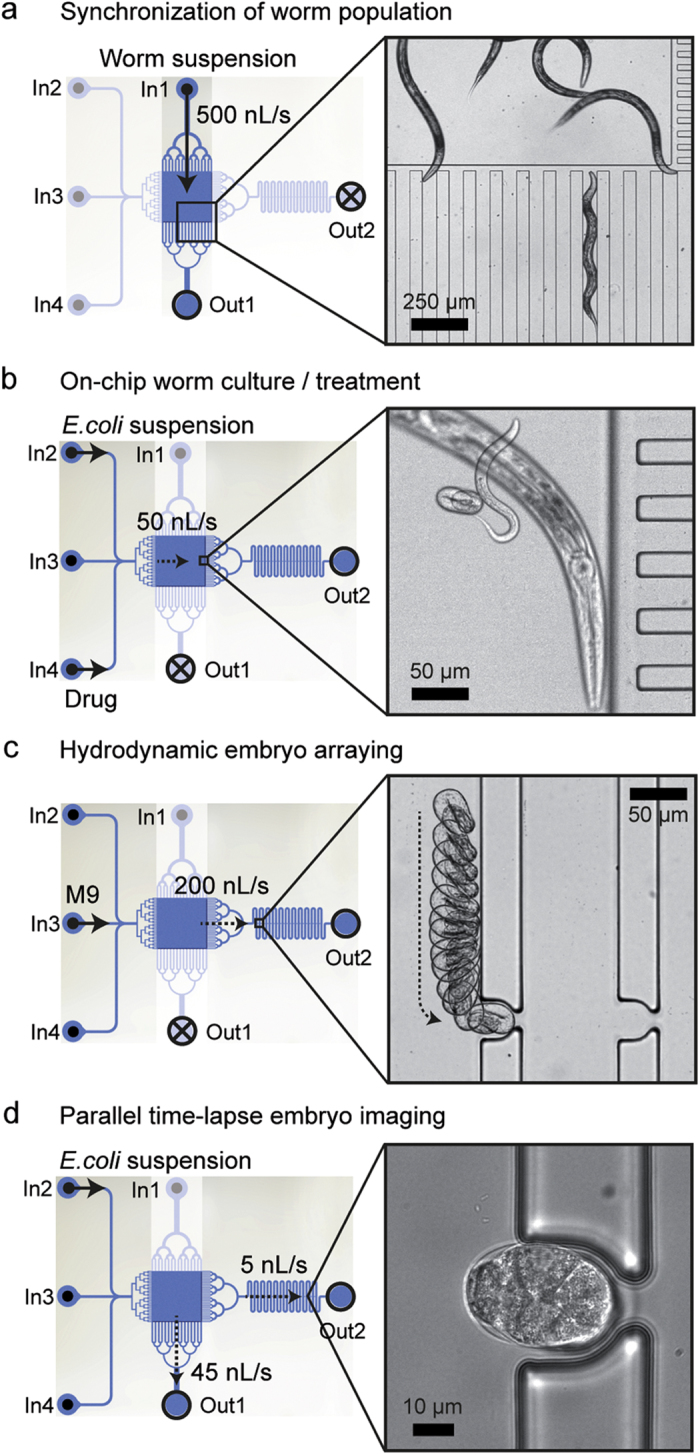
Operation of the microfluidic device. (**a**) First a 10 μL suspension of worms in M9 buffer is injected into the microfluidic device along the In1-Out1 direction at a flow rate of 500 nL/s. Symbols used for the in- and outlets: dot and arrow = syringe in use (e.g. In1); dot = syringe not in use (e.g. In2); circle = open valve (e.g. Out1); cross and circle = closed valve (e.g. Out2). The “worm synchronization filter” is tailored to retain inside the chamber only adult worms, as selected by their larger size and their better swimming abilities (see picture in zoom). (**b**) Subsequently worm culture is controlled by periodically injecting *E. coli* in M9 buffer along the In2-Out2 direction, typically at 50 nL/s flow rate. This ensures normal development of the worms in the liquid environment and continuous embryo production during their adult lifespan (see picture in zoom). Optionally, drugs or chemicals can be introduced at the In4 inlet for on-chip worm treatment. (**c**) Injection of M9 buffer at 200 nL/s flow rate along the In3-Out2 direction triggers the transfer of all the eggs present in the chamber towards the incubator array, where they are captured by passive hydrodynamics, as shown by the superposition of 13 video frames in the zoom. (**d**) The perfusion of *E. coli* suspension towards both Out1 and Out2 simultaneously ensures proper worm feeding inside the culture chamber and stable embryo positioning inside the incubators, enabling parallel time-lapse imaging of the embryos at cellular resolution (see picture in zoom).

In each experiment, worms and embryos are manipulated via sequences of fully automated operations (Fig. 2). The geometry of the chip is optimized for retaining inside the worm culture chamber only adult worms by washing out all the larvae present in a mixed worm suspension (zoom of Fig. 2a and Supplementary Video 2). Alternatively, a L1-L4 larvae suspension can be injected at a lower flow rate to retain just L4 larvae inside the chamber. Eventually, the number of captured worms can be adapted by running an optional “washing step” (Supplementary Video 3). Upon isolation of a defined worm population inside the chamber, worms are cultured and can be eventually treated on-chip with specific drugs or chemicals (Fig. 2b). An appropriate flow of M9 buffer allows recovering all the eggs present in the chamber and isolating each of them in a single micro-incubator via passive hydrodynamic trapping up to complete array filling (Fig. 2c and Supplementary Video 4). Parallel time-lapse imaging is then started, either for the full array or by scanning each individual embryo at high resolution, at desired frame rate, magnification and light wavelengths, depending on the analysis of interest (Fig. 2d).

### Automated analysis of embryonic morphogenesis

**Figure 3:**
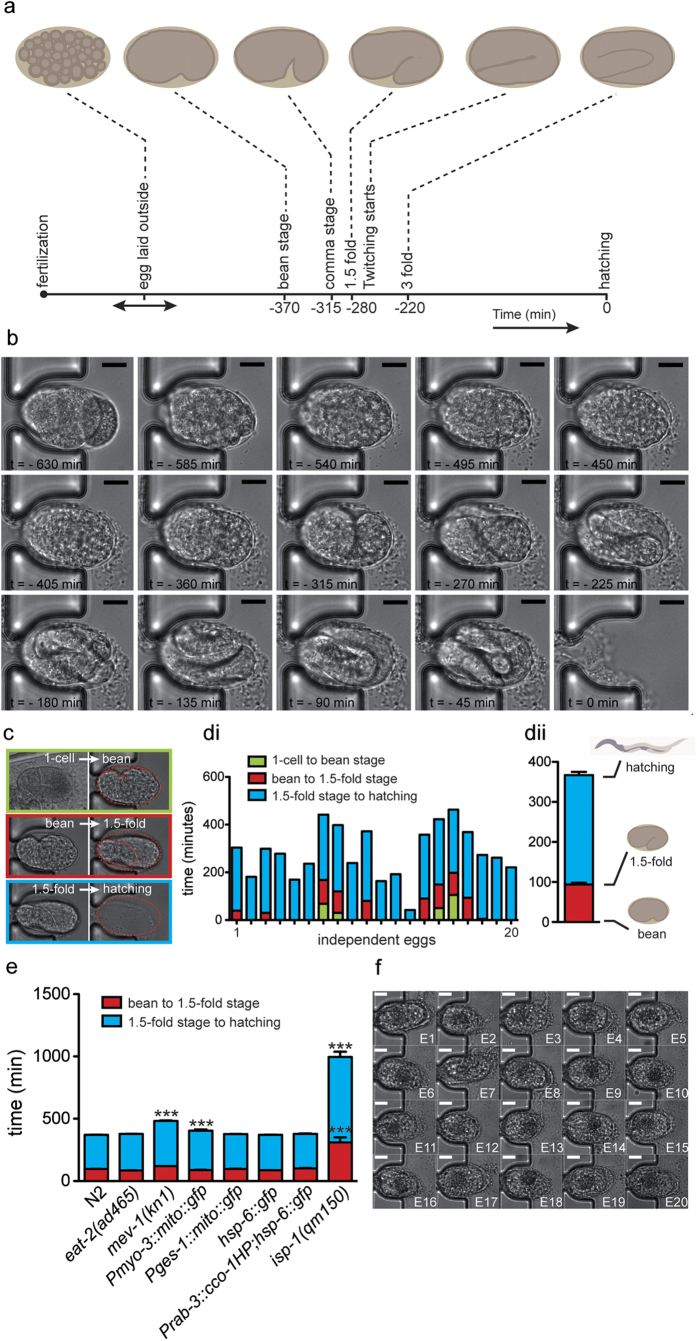
Study of *C. elegans* embryogenesis. (**a**) Time-lapse of the main embryonic stages of development with typical time indications for T = 25 °C and wild-type worms. (**b**) Full embryonic development from egg capture in the incubator till hatching, as observed in a sequence of brightfield microscopy images (63 × oil immersion objective, NA 1.4) taken from a movie (1 frame per minute) at 45 min intervals for a N2 wild-type worm strain at 25 °C; the hatching time defines t = 0. Scale bars=10 μm (**c**) Illustration of main embryonic development phases –1 cell to bean; bean to 1.5-fold; 1.5-fold to hatching– that are clearly morphologically distinguishable. (**d**) Duration of development phases, as observed for an array of 20 embryos for a N2 wild-type worm strain at 25 °C; (**d**i) variation of the time duration the embryo spends in an incubator, originating from differences in the exact moment of egg laying and trapping of the embryo; (**d**ii) average duration of development phases, as obtained from the data in (**d**i). (**e**) Duration of different development phases –bean to 1.5-fold; 1.5-fold to hatching– for the N2 wild-type strain of worms and a number of transgenic strains and mutants (Supplementary Note 6). Bar graphs are expressed as mean + SEM, *** *p* ≤ 0.001. (**f**) Pictures of a full array of 20 embryos taken 600 min after trapping in the incubators, illustrating the blocked development when the embryos are laid by N2 wild-type worms that were exposed in the culture chamber to 2 mM of the anticancer drug 5-fluorouracil (5-FU) in M9 buffer. Scale bars = 10 μm.

A microscopy environmental control system maintains a constant temperature on the chip (typically 25 °C) over the whole duration of each experiment. An automated xy-positioning stage is used to scan sequentially all positions of interest on the embryo incubator array. Embryos can be monitored at cellular resolution through a 63x, NA 1.4 oil immersion objective, thus allowing accurate observation and analysis of *C. elegans* embryonic morphogenesis stages (Fig. 3a) over the whole time-span, from egg capture to hatching (Fig. 3b and Supplementary Video 5). Two key events with clearly different morphological changes in the embryo shape can be distinguished: (i) the onset of the so-called “bean stage”, beginning of morphogenesis (Fig. 3c, top), and (ii) the “1.5-fold stage”, just before the twitching inception occurs (Fig. 3c, middle). Together with egg hatching (Fig. 3c, bottom), these morphological changes could be detected by software-controlled pattern recognition codes, for the full automation of the image processing.

We recorded the duration of the two last development phases of Fig. 3c for full arrays of embryos (n = 20) belonging to different strains (Supplementary Video 6). Our platform allowed accurate measurement of the duration of these phases for individual N2 wild-type embryos at 25 °C (Fig. 3di). The apparent variability is an indication of variations in the exact moment of egg laying (and subsequent trapping) of each embryo, which represents another interesting phenotype to be studied with our method as well. The average duration of the development phases could be monitored with good accuracy, even from this single array experiment (Fig. 3dii). Moreover, as worms could be cultured and maintained on the same chip for several days, we demonstrated the capability of our device to be employed for studying age-related changes in worm reproduction and progeny development (Supplementary Note 5).

We systematically studied the duration of the different development phases for several transgenic strains and mutants (Fig. 3e and Supplementary Note 6). As for the wild-type, we could reliably determine the average duration of the bean to 1.5-fold stage phase (phase 2) (~90 min) and the average duration of the 1.5-fold stage to hatching phase (phase 3) (~280 min) in the *eat-2(ad465), Pges-1::mito::gfp, hsp-6::gfp and Prab-3::cco-1HP;hsp-6::gfp* strains. A weak increase of the phase 3 was measured with the *Pmyo-3::mito::gfp* transgenic strain, which is in accordance with the slight developmental delay observed during the larval stage (data not shown). A more significant 1.3-fold increase of phase 3 is instead observed in the *mev-1(kn1)* mutants, while for the *isp-1(qm150)* mutants, both phases 2 and 3 were more than two times longer compared to the control worms. These results already highlight the importance of the mitochondrial electron transport chain function during embryogenesis (Supplementary Note 6).

Eventually, the possibility of automated on-chip chemical or drug treatment was validated by exposing the worms to the anticancer drug, 5-fluorouracil (5-FU). This compound induces cell-cycle arrest and apoptosis of germ-line cells in *C. elegans*^16^. In our experiment, wild-type worms were isolated in the culture chamber at the L4 larval stage and treated on-chip with 5-FU at a concentration of 2 mM, while being cultured at 25 °C towards the adult stage. After washing the chamber with clean M9 buffer, successively laid embryos were transferred to the incubator array and monitored for 12 h. All embryos prematurely died, proving the efficiency of the drug exposure of the worms in the culture chamber of the chip (Fig. 3f).

### Study of mitochondrial biogenesis during embryonic development

**Figure 4:**
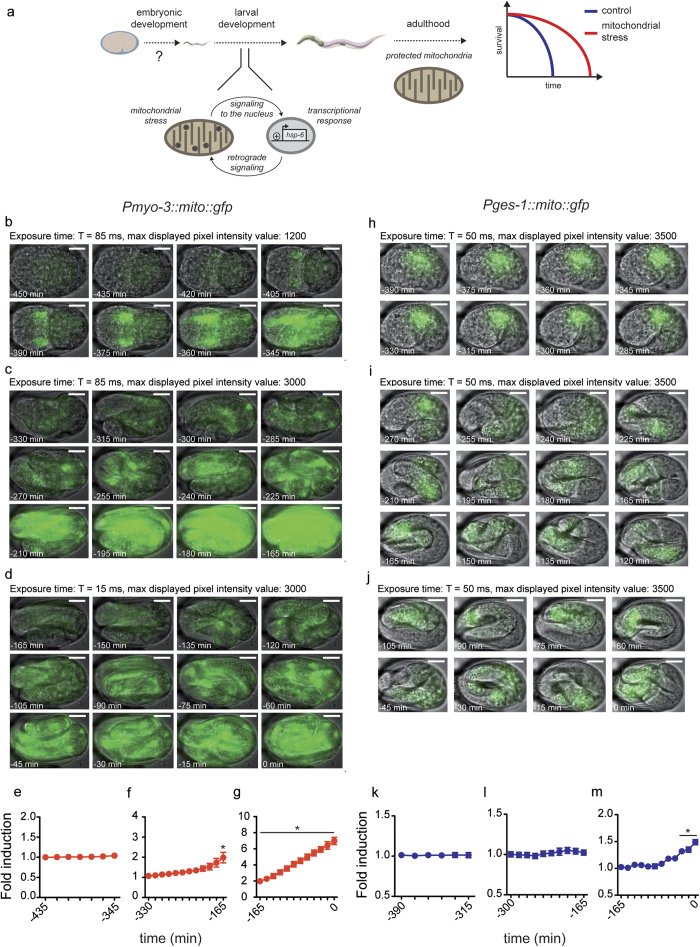
Study of mitochondrial biogenesis. (**a**) Schematic representation of the mitochondrial unfolded protein response (UPR^mt^), as known for the larval and adult stages of the nematode *C. elegans*. (**b**-**d**) Merges of optical brightfield and fluorescent pictures of the *Pmyo-3::mito::gfp* embryos and (e–g) the corresponding GFP quantification over the whole time-span from embryo capture to hatching (n = 4). Pictures are grouped in blocks of same experimental observation and image representation conditions (exposure time and maximum displayed pixel intensity). (**h**-**j**) Merges of optical brightfield and fluorescent pictures of the *Pges-1::mito::gfp* embryos and (k–m) the corresponding GFP quantification over the whole time-span from embryo capture to hatching (n = 14). Pictures are grouped in similar blocks as for the *Pmyo-3::mito::gfp* embryos. For (**e**-**g**) and (**k**-**m**), the curves correspond to the relative GFP induction compared to the initial time point in (**e**) and (**k**), respectively, of the experiments. Bar graphs are expressed as mean + SEM, * *p* ≤ 0.05, points below a horizontal line are significantly different from the corresponding control. Scale bars = 10 μm.

Knowing that the knockdown of many mitochondrial genes can induce embryonic lethality or result in an infertile phenotype, we investigated mitochondrial function and biogenesis during embryogenesis, focusing on the molecular pathway known as mitochondrial unfolded protein response (UPR^mt^). The latter is an adaptive response that monitors and subsequently repairs abnormal proteostasis within the mitochondria and, as such, ensures the proper function and integrity of the mitochondria^17^,^18^. In *C. elegans*, it has been shown that UPR^mt^ is most effective when triggered during larval development, resulting in a permanent adaptive protection of the mitochondria throughout life and, as such, increasing the animal’s survival rate (Fig. 4a). This careful control of mitochondrial activity early in life seems to setup a proper rate of aging, which persists throughout life, probably through the establishment of epigenetic imprints^19^. However, it is still unknown whether the UPR^mt^ is operational during the earliest phases of the life, *i.e.* embryonic development. As tackling this problem requires the careful analysis of multiple parameters in a spatio-temporal controlled fashion in a living embryo, we used our platform to address this challenging question.

Before measuring the UPR^mt^ activity, we first investigated whether mitochondrial biogenesis could be detected and monitored in two different tissues in embryos, i.e. the muscle and the intestine. Indeed, a proper mitochondrial proliferation and activity is a prerequisite for the induction of a mitochondrial stress. Two transgenic strains of worms were used, expressing Green Fluorescent Protein (GFP) either in the mitochondria of the body wall muscle cells, i.e. the *Pmyo-3::mito::gfp* worm strain SJ4103, or in the intestinal cells, i.e. the *Pges-1::mito::gfp* worm strain SJ4143^20^. Larvae or adult worms of these strains are commonly used to monitor the mitochondrial activity, proliferation and morphology^21^,^22^. Brightfield and fluorescent pictures of the same embryo were quasi-simultaneously recorded and superimposed, permitting accurate localization of the fluorescent signal. Our analysis revealed that mitochondrial proliferation takes place in both muscle and intestinal cells (Fig. 4b–g and Supplementary Video 7, Fig. 4h–m and Supplementary Video 8). Interestingly, the mitochondrial biogenesis looks different in terms of intensity between muscle and intestinal cells, probably due to the different number and role of mitochondria, as it is clear when comparing Fig. 4b–d with Fig. 4h–j. Indeed, after the 1.5-fold stage of embryogenesis, most cellular proliferation and migration of the body wall muscles are completed and the worm starts twitching in the eggshell, requiring a sustained energy supply provided by the mitochondrial activity^23^. Beside the intensity of mitochondrial biogenesis during embryogenesis, our study revealed that mitochondrial proliferation behaves independently of tissue morphogenesis. While genetic markers of the body wall muscle and the intestinal cells significantly appear before the bean stage^24^, we observed a rather large delay regarding the onset of mitochondrial proliferation, highlighting a specific increase of the energy supply during the late phase of the embryo development (Fig. 4e–g and Fig. 4k–m). Furthermore, as already observed in mouse embryos, this late proliferation of mitochondria could coincide with the pronounced structural and functional differentiation of the mitochondria within the different tissues^25^. Interestingly, a previous study showed that the mitochondrial DNA copy number –which is indicative of the relative number of mitochondria in the organism – remains unchanged from the late embryo stage up to the L3 larval stage, whereas a five-fold increase occurs with the transition from L3 to L4^26^. Our experimental observations suggest that a first increase in mitochondrial biogenesis actually takes place earlier, during the embryogenesis (Fig. 4g,m). Although embryonic mitochondrial biogenesis is less pronounced compared to that occurring during the L3 to L4 transition, it is significant and we speculate that it provides the necessary amount of mitochondria and energy, required for the first steps of the larval development.

### Study of mitochondrial unfolded protein response during embryogenesis

**Figure 5:**
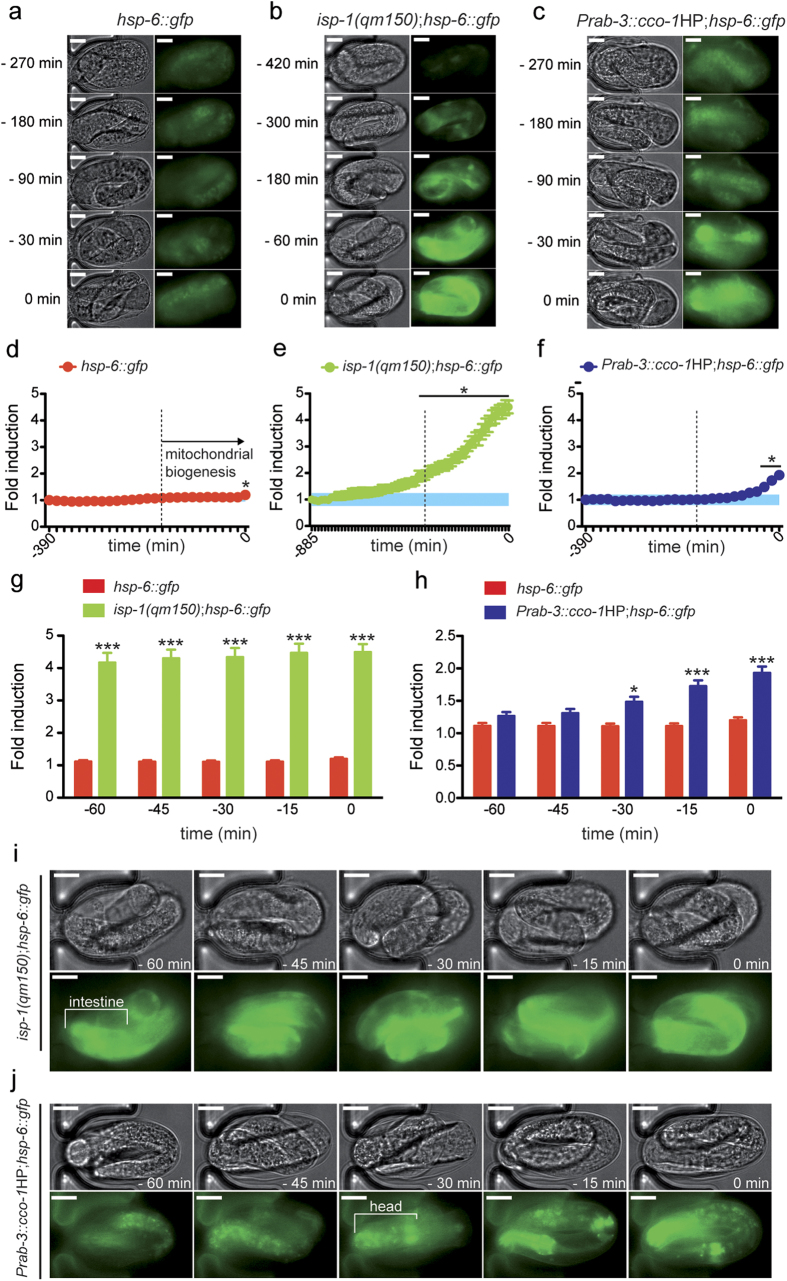
Study of mitochondrial unfolded protein response (UPR^mt^) during embryogenesis. (**a**-**c**) Representative optical brightfield and corresponding fluorescent pictures that show the *hsp-6::gfp* expression in (**a**) wild type, (**b**) *isp-1(qm150)*, and (**c**) *Prab-3::cco-1HP* strains. (**d**-**f**) Quantification of the *hsp-6::gfp* induction in (**d**) wild type, (**e**) isp-1(*qm150*) and (**f**) *Prab-3::cco-1HP* strains over the whole time-span from embryo capture to hatching (n = 17, 7, and 21, respectively). The curves correspond to the relative GFP induction compared to the initial time point of each experiment. The blue area in each graph represents the base level 95% interval of confidence for each experiment. Bar graphs are expressed as mean+SEM, * *p* ≤ 0.05, points below a horizontal line are significantly different from the corresponding control. (**g**-**h**) Comparison of the relative wild type *hsp-6::gfp* induction with the one of the (**g**) *isp-1(qm150)* and (**h**) *Prab-3::cco-1HP* strains. Bar graphs are expressed as mean+SEM, * *p* ≤ 0.05, *** *p* ≤ 0.001. (**i**-**j**) Localization of the *hsp-6::gfp* expression in the embryonic tissues, as observed in a period starting 60 min before hatching, for the (**i**) *isp-1(qm150)* and (**j**) *Prab-3::cco-1HP* strains. Scale bars = 10 μm.

We then investigated whether the UPR^mt^ can also be detected during embryogenesis. To monitor the mitochondrial stress response in living embryos, we used a transgenic strain of worms that reports on the activity of the UPR^mt^ with integrated GFP genes driven by the regulatory DNA region of the mitochondrial chaperone *hsp-6*^31^. In these transgenic worms, an increase of *hsp-6::gfp* expression is indicative of the presence of a mitochondrial stress and the subsequent induction of the UPR^mt^ 28,29. We first examined the UPR^mt^ induction in unstressed embryos. Despite a pronounced mitochondrial proliferation occurring during the late phase of the embryogenesis, we did not observe a matching increase of *hsp-6::gfp* expression (Fig. 5a,d). This result is in agreement with previous reports showing an activation of the UPR^mt^ during the L3 to L4 transition period, where a similar burst of mitochondrial biogenesis occurs only if a mitochondrial stress is experimentally induced^26^,^28^. Nevertheless, a careful monitoring and surveillance of mitochondrial stress is fundamental for the embryogenesis, as the loss of function of some UPR^mt^ mediators, such as *hsp-6* and *dve-1*, has been reported to lead to an early death of the worm embryos^30^,^31^. To trigger a potential constitutive UPR^mt^ in embryos, we first used the mutant strain MQ887 carrying a mutation in the *isp-1* gene, which we crossed with the *hsp-6::gfp* worms. The resulting *isp-1(qm150);hsp-6::gfp* strain showed a constitutive activation of *hsp-6::gfp* in larvae and adult worms, revealing a strong and continuous mitochondrial stress^28^ (Supplementary Note 7). By monitoring the *hsp-6::gfp* expression during the embryo development, we observed a 4- to 5-fold induction of the UPR^mt^, which matches the onset of mitochondrial biogenesis (Fig. 5b,e,g and Supplementary Video 9). Interestingly, this mitochondrial stress response seems to be more pronounced in the intestinal cells, which is in accordance with previous observations in larvae and adults and confirms that this tissue is one of the prime sites for the activation of the UPR^mt^ (Fig. 5i)^27^.

While UPR^mt^ can be activated in a cell-autonomous manner, as here illustrated in the intestine, this stress response can also be triggered in a cell-non-autonomous manner^28^. To explore this hypothesis, we used the *hsp-6::gfp* strain of worms carrying an additional transgene, which drives the knockdown of the mitochondrial gene *cco-1* only in neurons, i.e. the *Prab-3::cco-1HP;hsp-6::gfp* worm strain AGD1073 (Supplementary Note 8). In the post-embryonic phase of worm development, the silencing of *cco-1* exclusively in neurons is sufficient to promote a mitochondrial stress that is propagated in distal tissues^28^. We observed a robust 2-fold induction of UPR^mt^ when *cco-1* is silenced in neuronal cells during the embryogenesis (Fig. 5c,f,h and Supplementary Video 10). Interestingly, the activation of the mitochondrial stress response seems independent of the *rab-3* promoter activity –*rab-3* starts being expressed in the early cell division stages^24^ – but rather a consequence of the concomitant mitochondrial proliferation and *cco-1* knockdown. Furthermore, while the UPR^mt^ induction takes place in the intestinal cells of the *isp-1(qm150)* embryos (Fig. 5i), neurons seem to be the primary cells for the mitochondrial stress response in the case of *cco-1* silencing in the neuronal system (Fig. 5j). This difference between embryos and larvae concerning the lack of cell-non-autonomous activation of UPR^mt^ could be explained by the fact that the neuronal and hormonal signalling pathways required for signal transmission are not yet fully developed at the embryonic stage.

## Discussion

In our platform, a well-defined and synchronized *C. elegans* embryo population can be isolated from an on-chip worm culture and studied in a fully automated way at extremely high spatial and temporal resolution. The device allows operation and analysis at the single-organism level, thus preserving the identity of each individual embryo, while at the same time providing statistics of the complete population. We demonstrated the capability of our platform to accurately analyze the real-time dynamics of different phases of the embryonic development, to monitor live protein expression in developing embryos during the complete embryogenesis, and to perform systematic studies that address outstanding issues in developmental biology.

Our approach allows suppressing the bleaching step in the classical method, which is used for embryo harvesting and could affect the embryo integrity. Furthermore, our on-chip embryo synchronization allows using a minimal amount of gravid worms to obtain an accurate number of laid eggs. Finally, the limitation of the manual handling and the full automation of our protocol provide great advantages compared to the actual standard procedure, requiring several manual preparation steps (maintenance of a large worm population on solid agar plate, recovery of this worm population with the bleaching solution, washing of the egg preparation, egg transfer, etc.).

In particular, we validated our platform by characterizing development, mitochondrial biogenesis and UPR^mt^ in worm embryos at a precision that would have been very difficult or impossible to achieve with classical worm techniques. The imaging and monitoring of the embryogenesis require specific techniques, which imply single embryo isolation and its mounting^2^. Our microfluidic device is specifically designed to simplify and automate embryo handling, and only requires loading of a few adult worms into the chip. Furthermore, classical protocols for embryo analysis do not allow the concomitant monitoring of multiple replicates in identical biological conditions. The incubator array format of this platform provides a unique opportunity to study the intra-embryo variability in terms of viability, development and gene expression. This platform allows the identification in a reproducible and accurate manner of the different phases of the *C. elegans* embryogenesis that occur after the egg laying, from the early cell division stages to hatching. We were able to discriminate variations in terms of embryonic development and describe how perturbations of the mitochondrial functions can have a profound impact on the embryogenesis.

In *C. elegans*, the mitochondrial biogenesis mainly occurs during the late larval phase, *i.e.* during the L3 to L4 larval transition^26^. Here we described that another burst of mitochondrial biogenesis takes place during the last phase of the embryo development. This late embryonic proliferation of mitochondria seems to overlap with the differentiation of the mitochondria within the tissues, as revealed by the different profiles in term of biogenesis intensity and timing observed in muscle and intestinal tissues. Furthermore, while the mitochondrial biogenesis that occurs during the embryogenesis is less intense compared to the L3 to L4 transition, it is tempting to speculate that this late event in the embryo could be a prerequisite for the first steps of the larval development, by providing the required amount of mitochondria and energy. The crucial importance of the late phase of embryonic mitochondrial biogenesis is corroborated by the observation that several disturbances of the mitochondrial function during this specific period can impact later in worms’ life^32^.

In addition, we demonstrated that such perturbations of the mitochondrial function can trigger the UPR^mt^ pathway in embryos. The activation of this specific mitochondrial stress response matches the onset of mitochondrial biogenesis, which is in accordance with what is observed in larvae. Furthermore, the UPR^mt^ seems to work mainly in a cell autonomous manner in embryos, probably because the signalling pathways required for the signal transmission between neurons and the distal tissues are not yet fully developed at this stage of life. These observations are the first evidence that UPR^mt^ is functional during the embryogenesis. One could hypothesize that a mitochondrial stress restricted to the late phase of the embryo development could trigger a beneficial effect during the rest of the life, opening the concept of a potential “mitochondrial imprinting” during the first step of the organism’s life.

In the future, due to the versatility of our platform design, its live imaging capability can be readily extended to include other types of microscopies, like differential interference contrast microscopy, for high-contrast brightfield live imaging, and confocal microscopy to achieve extreme spatial resolution. Computer-enhanced image processing can be used to further extend the analytical possibilities of our platform for real-time embryonic screening and phenotyping, or even automated cell lineage and expression profiling in the developing embryos. In our device embryos are isolated immediately after they are naturally laid, thus their monitoring typically starts at the 26 to 44-cell stage. However, with our system, earlier cell division events may be optionally observed by directly injecting embryo suspensions prepared via standard manual bleaching protocols into the device. As in our platform worms are directly cultured on-chip and embryos analyzed upon spontaneous egg-laying, the whole information related to the natural reproduction process is preserved, maintaining the link between parents and progeny. Therefore, the platform is directly suitable for investigating trans-generational properties on the embryos and, with some adaptation of the microfluidic design, even studying the progeny and epigenetic imprints in successive worm generations. Devices for related parasitic nematodes can be readily designed by re-adapting the incubator size, for example to study the effects of anti-parasitic drugs. Finally, we expect that similar microfluidic designs will be used to perform live imaging of a multitude of development events, like gastrulation and tissue morphogenesis during embryogenesis in other species of nematodes or other model organisms.

## Methods

### Chemicals and Materials

4-inch 550 μm thick Si and float glass wafers, de-ionized water (DIW) were obtained from the Center of Micro- and Nanotechnology of EPFL. GM 1070 SU-8 negative photoresist was purchased from Gersteltec (Pully, Switzerland). PDMS Sylgard 184 was acquired from Dow Corning (Wiesbaden, Germany). 1 mL borosilicate H-TLL-PE syringes were purchased from Innovative Labor Systeme (Stutzerbach, Germany). Microline ethyl vinyl acetate tube with 0.51 mm inner and 1.52 mm outer diameters was bought from Fisher Scientific (Wohlen, Switzerland). Pluronic F-127 was purchased from Sigma-Aldrich (Buchs, Switzerland). M9 buffer was obtained by adding 3 g KH_2_PO_4_, 6 g Na_2_HPO_4_, 5 g NaCl, 1 mL 1 M MgSO_4_, H_2_O to 1 litre and sterilization by autoclaving. Pluronic F127 solution was prepared by diluting 0.02% (weight/volume) Pluronic F127 in M9.

### *C. elegans* strains and culture

*C. elegans* strains were cultured at 20 °C on nematode growth media agar plates seeded with *Escherichia coli* strain OP50, unless stated otherwise. Strains used were wild-type Bristol N2, DA465 *eat-2(ad465)* II, MQ887 *ips-1(qm150)* XX, SJ4100 (zcIs13[*hsp-6*::GFP]), SJ4103 (zcIs14[*myo-3*::GFP(mit)]), SJ4143 (zcIs17[ges-1::GFP(mit)]), TK22 *mev-1(kn1)*. Strains were provided by the Caenorhabditis Genetics Center (University of Minnesota). The AG1073 (*Prab-3::cco-1HP;hsp-6::gfp*) strain of worms was kindly provided by Andrew Dillin (UC Berkeley, CA, USA). Worms were suspended in M9 solution prior to each microfluidic experiment. Microfluidic worm synchronization procedures are detailed in Fig. 2.

### Fabrication of the microfluidic chips

Microfluidic devices were prepared by soft lithography^33^ using 2-layer SU-8 molds. Briefly, conventional photolithography was used to pattern a 35 μm-thick layer of SU-8 photoresist on 4-inch wafers. A 85 μm-thick layer of SU-8 was then patterned on top of the first one. The silicon mold was then diced in 15 mm × 18 mm microchips, which were inserted at the bottom of an aluminum/polymethylmetacrylate (PMMA) mold for PDMS casting. 1.5 mm diameter steel pins were used to define the lateral connections of the device for the external tubing insertion. A liquid PDMS mixture (10:1 base:cross-linker weight ratio) was degassed, injected into the mold and cured at 100 °C for 1 h. Upon extraction from the mold, each PDMS chip was bonded by plasma-activation to a 150 μm-thick glass coverslip. The chip was then connected to external tubing and enclosed in a PMMA holder (Fig. 1a), designed for the observation of the device through any upright or inverted microscope and with any kind of objective.

### System control and microfluidic device operation

A live-cell microscopy environmental control system (Visitron, Puchheim, Germany) allowed controlling the chip temperature over the whole duration of each experiment. The microfluidic operations were controlled using Nemesys syringe pump control software (Cetoni, Korbussen, Germany). Experimentally, the microfluidic chip was first filled with Pluronic F127 solution, incubated for 30 min inside the device, to prevent *E. coli* sticking and accumulation inside the microchannels^34^. Few worms from a non-synchronized population were suspended in 10 μL of M9 buffer and sucked in a microfluidic tube, which was then connected to the device. From this point on, the system was completely controlled by software, through the automated sequential steps described in Fig. 2.

### Automated operation of the platform

In each experiment, worms and embryos are manipulated via sequences of fully automated operations (Fig. 2). A worm suspension is first injected into the microfluidic device through the top port (In1 of Fig. 2a) and directed towards the “worm synchronization filter” by opening the valve at “Out1”. The geometry of the chip is optimized for retaining inside the worm culture chamber only adult worms by simply selecting the correct flow rate for the sample injection. In practice, at a flow rate of 500 nL/s, in a few tens of seconds, all the larvae present in the suspension are directly washed out of the chip, while adult worms are kept inside the chamber both due to their larger size and their better swimming abilities/resistance against the flow (zoom of Fig. 2a and Supplementary Video 2). The number of worms retained inside the chamber is controlled by the concentration of young adults in the worm suspension injected into the device (typically, 5 to 10 young adults per 10 μL suspension). Alternatively, a L1-L4 larvae suspension can be injected at a flow rate of 100 nL/s to retain just L4 larvae inside the chamber. When needed, the number of captured worms can be adapted by running an optional “washing step”, where M9 buffer is injected for a few seconds along the In1-Out1 direction at higher flow rates (1 to 5 μL/s) (Supplementary Video 3). Upon isolation of a defined worm population inside the chamber, worms are cultured and can be eventually treated on-chip with specific drugs or chemicals (Fig. 2b). For worm culture/treatment, an *E. coli* suspension is injected in the chamber at a desired rate, through the In2-Out2 direction, while drugs or chemicals can be introduced in the chip at controlled concentration and precise instants of the worms’ lifespan. A simple increase of the flow speed inside the chamber along the In3-Out2 direction is then used to transfer the eggs present in the chamber towards the embryo-incubator array. In practice, a 200 nL/s flow of M9 buffer allows recovering all the eggs present in the chamber and isolating each of them in a single micro-incubator via passive hydrodynamic trapping up to complete array filling (Fig. 2c and Supplementary Video 4). Parallel time-lapse imaging is then started, either for the full array or by scanning each individual embryo at high resolution, at desired frame rate, magnification and light wavelengths, depending on the analysis of interest (Fig. 2d). Alternatively, parallel time-lapse imaging could be started at the beginning of the embryo collection phase, in order to monitor each embryo from the first moment of its trapping on. Brightfield imaging of the 20 trapped embryos takes 20 seconds, and this procedure is repeated every minute. In the fluorescence experiments, combined brightfield and fluorescent imaging of the 20 trapped embryos is done in 180 seconds, and this procedure is repeated every 15 minutes, to avoid phototoxicity effects on the embryos. The synchronization point in the development for all embryos can be chosen by using either the appearance of the bean stage or hatching. During live imaging, we apply a slow flow (5–10 nL/s) of M9 buffer along the In3-Out2 direction to assure stable positioning of the embryos in the array. Because of the large section difference between chamber and incubators, the flow speed is slower inside the culture chamber and this very gentle flow is strong enough to keep captured eggs in position, but not fast enough to transfer new eggs from the chamber to the array. Optionally, for sequential studies on embryo populations produced by the same worms at different periods of their full adult lifespan, worm culture can be maintained in the chamber by the perfusion of *E. coli* from In2 inside the chip. Both the valves at Out1 and Out2 are left open in this case, and the different hydrodynamic resistances of the two orthogonal directions result in a partitioning of the flow between the two outlets, with most of the liquid flowing through Out1. This establishes a slow flow through the incubator array, ensuring stable positioning of the embryos over long periods, while still reducing *E. coli* accumulation in the array area, which could compromise the results of embryo fluorescent imaging, because of the autofluorescence of *E. coli* bacteria.

### Image acquisition and processing

The microfluidic chip was integrated onto an inverted microscope (Axio Observer, Zeiss) equipped with two illumination systems: (i) a precisExcite High-Power LED Illumination system (Visitron, Puchheim, Germany) for brightfield imaging and (ii) a Lambda DG4 illumination system (Sutter instruments, Novato, CA, USA) for fluorescence imaging. The microscope had a motorized xy-stage, equipped with an ASI piezo controller for z-displacement (Visitron, Puchheim, Germany) and the automated imaging process was controlled using VisiView Premier Image acquisition software (Visitron, Puchheim, Germany).

To start the automated imaging process, the position of the first egg in the array was set as initial point of the xy-stage scanning, while the locations of the other eggs were automatically determined by the interdistance between adjacent incubators (118 μm). A “wavelength program” was set on the software, to automatically switch between brightfield and fluorescent imaging modes, by controlling both the illumination systems. “Time-lapse” and “stage position” programs were set to automatically perform scanning and imaging over the full array at a desired rate, hence resulting in parallel time-lapse imaging of all the embryos. To avoid phototoxicity effects during these fluorescence imaging experiments, we minimized the exposure time of the embryo to the fluorescent excitation light (t < 100 ms) and pictures were recorded at a single focal plane of the microscope (i.e. at a single z value), despite the possibility of taking z-stacks with our setup. The movement of the embryo inside its eggshell during the twitching phase could sometimes introduce instantaneous modulations in the collected fluorescent intensity, because of the time-dependent positioning of the developing larva inside the focal volume of the microscope objective, but variations of the average fluorescent intensity of the embryo in a given xy-plane remained relatively small. The fluctuations in fluorescent intensity due to twitching of the embryo are smaller than the error bars in Figs. 4,5. For each fluorescent picture, GFP intensity values were measured as average pixel intensity over the area occupied by the single embryo under observation. A background value was then measured for each picture as average pixel intensity over the microchannel area and this value was subtracted from the previously calculated one, to exclude the influence of any external autofluorescence sources from the measurement. Moreover, unspecific GFP expression is avoided by the use of tissue-specific promoters, e.g. the *myo-3* promoter triggers GFP expression in the body wall muscles, while the *ges-1* promoter in the intestinal cells only.

A simple Matlab script (MathWorks, Natick, MA, U.S.A) was written to reorder the large amount of data of each experiment according to the image xy-coordinates, time, light wavelength and exposure time. Image processing was performed with Fiji software ( http://imagej.nih.gov/ij; version 1.47b).

## Additional Information

**How to cite this article**: Cornaglia, M. *et al*. An automated microfluidic platform for *C.elegans* embryo arraying, phenotyping, and long-term live imaging. *Sci. Rep.* **5**, 10192; doi: 10.1038/srep10192 (2015).

## Supplementary Material

Supporting Information Supplementary Notes

Supporting Information Supplementary Video 1

Supporting Information Supplementary Video 2

Supporting Information Supplementary Video 3

Supporting Information Supplementary Video 4

Supporting Information Supplementary Video 5

Supporting Information Supplementary Video 6

Supporting Information Supplementary Video 7

Supporting Information Supplementary Video 8

Supporting Information Supplementary Video 9

Supporting Information Supplementary Video 10

Supporting Information Supplementary Video 11

Supporting Information Supplementary Video 12
