# Supplementary material for: An automated microfluidic platform for C. elegans embryo arraying, phenotyping, and long-term live imaging
Source: Sci Rep. 2015 May 7;5:10192. doi: 10.1038/srep10192 (PMC4423638; doi:10.1038/srep10192)
Supplement: Supporting Information — Supplementary Notes [file srep10192-s1.pdf]

Supplementary Information

An automated microfluidic platform for *C.elegans* embryo arraying, phenotyping, and long-term live imaging

Matteo Cornaglia[1], Laurent Mouchiroud[2], Alexis Marette[1], Shreya Narasimhan[1], Thomas Lehnert[1], Virginija Jovaisaite[2], Johan Auwerx[2], and Martin A. M. Gijs[1]*

[1]Laboratory of Microsystems, EPFL, CH-1015 Lausanne, Switzerland

[2]Laboratory for Integrative and Systems Physiology, EPFL, CH-1015 Lausanne, Switzerland

Supplementary Notes

**Supplementary Note 1**	Detailed geometrical description of the worm culture chamber.
**Supplementary Note 2**	Optimization of the hydrodynamic trapping system.
**Supplementary Note 3**	Optimization of the embryo-incubator geometry.
**Supplementary Note 4**	Analysis of embryo positioning inside the incubators.
**Supplementary Note 5**	Age-related changes in worm reproduction and progeny.
**Supplementary Note 6**	Duration of the different development phases for transgenic strains and mutants.
**Supplementary Note 7**	Mitochondrial stress in the *isp-1(qm150);hsp-6::gfp* transgenic strain at the adult stage.
**Supplementary Note 8**	Mitochondrial stress in the *Prab-3::cco-1HP;hsp-6::gfp* transgenic strain.

Supplementary Videos, as available on the journal's website.

**Supplementary Video 1**	Microfluidic control of the hydrodynamic trapping mechanism of *C.elegans* embryos
**Supplementary Video 2**	Worm injection into the culture chamber and population synchronization at the adult stage
**Supplementary Video 3**	Complete washing of the worm culture chamber
**Supplementary Video 4**	Microfluidic transfer of a single *C.elegans* embryo from the worm culture chamber to the first empty embryo-incubator
**Supplementary Video 5**	Development of a wild-type *C. elegans* embryo within an embryo-incubator at 25°C, from its first cellular divisions until hatching (brightfield microscopy, 63x NA 1.4 objective)
**Supplementary Video 6**	High-resolution parallel imaging of a full array of 20 wild-type *C.elegans* embryos, from array filling to hatching of the last alive embryo (brightfield microscopy, 63x NA 1.4 objective)
**Supplementary Video 7**	Mitochondrial biogenesis in the body wall muscle cells of a *C.elegans* developing embryo, as observed in the *Pmyo-3::mito::gfp* transgenic strain (merge of brightfield and fluorescent pictures, 63x NA 1.4 objective)
**Supplementary Video 8**	Mitochondrial biogenesis in the intestinal cells of a *C.elegans* developing embryo, as observed in the *Pges-1::mito::gfp* transgenic strain (merge of brightfield and fluorescent pictures, 63x NA 1.4 objective)
**Supplementary Video 9**	Mitochondrial stress response (UPR^{mt} activation), as observed in a developing embryo of the *isp-1(qm150);hsp-6::gfp* mutant strain (merge of brightfield and fluorescent pictures, 63x NA 1.4 objective)
**Supplementary Video 10**	hsp-6::gfp expression in developing embryos of the *hsp-6::gfp* strain and the *Prab-3::cco-1HP;hsp-6::gfp* strain (merge of brightfield and fluorescent pictures, 63x NA 1.4 objective)
**Supplementary Video 11**	Effect of high fluidic pressures on worms and embryos inside the microfluidic device
**Supplementary Video 12**	Complete washing of the embryo-incubator array by single flow pulse

Supplementary Note 1. Detailed geometrical description of the worm culture chamber.

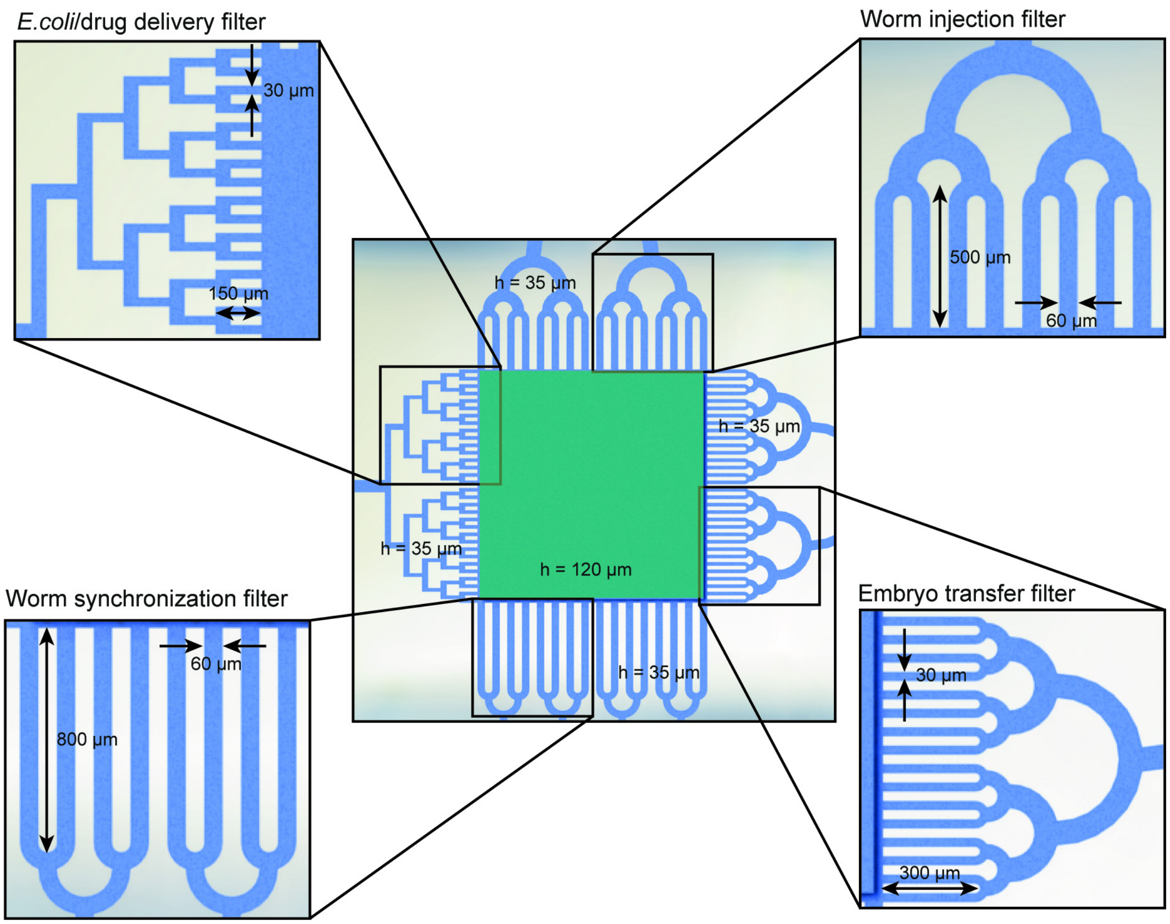

Figure SI1 | Geometry of the worm culture chamber. Schematic representation of the worm culture chamber, with zooms on the surrounding microfluidic channel arrangements and their specific dimensions.

Supplementary Note 2. Optimization of the hydrodynamic trapping system.

The efficiency of the hydrodynamic trapping mechanism in the embryo incubator can be estimated by calculating the ratio between Q_1 and Q_2, i.e. the volumetric flow rates through each incubator and its bypass channel, respectively (Figure SI2ai). This can be done by first considering the pressure drop between points A and B, along the two different fluidic paths and then imposing equal pressure drop along them (ignoring minor losses due to channel bends, widening/narrowing, etc.)[1]. At steady-state flow conditions, for incompressible Newtonian fluids flowing through straight microchannels (i.e. Poiseuille flow), this operation actually corresponds to calculating the inverse of the ratio between the hydraulic resistances of the two paths, R_2/R_1. For straight channels with rectangular cross-sectional shape, the hydraulic resistance can be calculated as:[2]

$$R = \frac{12\eta L}{1 - 0.63\,(\frac{h}{w})}\frac{1}{h^3 w} \qquad \text{(S1)}$$

where η is the fluid viscosity, L the channel length, h and w the channel height and width, respectively.

It has been shown that, for spherical objects (e.g. micro-beads), good trapping efficiencies are achieved for ratios $Q_1/Q_2 = R_2/R_1 > 3$.[1] However, no data were available on the hydrodynamic trapping of non-spherical objects for an incubator/bypass microfluidic design. Hence, we first estimated the trapping efficiency of our device for different array geometries and then fabricated two microfluidic devices with different Q_1/Q_2 ratios to experimentally evaluate their capturing efficiency for *C.elegans* embryos. The efficiency E is defined as the number of experimentally captured embryos over the total amount of embryos injected into the microfluidic device.

Table SI1. Geometrical parameters, as defined in Figure SI2aii, Q_1/Q_2 **ratio, and experimental embryo capture efficiency of two microfluidic designs.**

	W_c (μm)	L_c (μm)	W_i (μm)	L_i (μm)	W_d (μm)	L_d (μm)	Q_1/Q_2	*E* (%)
**Design A**	100	1000	35	50	12	8	~4	~50
**Design B**	80	1200	35	30	12	8	~13.5	~100

The geometrical parameters of table SI1, as defined in Figure SI2aii, have been chosen following specific needs related to the capture of *C.elegans* embryos. For instance, the bypass channel width has to be large enough to allow the passage of embryos at any orientation and avoid channel clogging, even when a group of embryos is flowing through the serpentine. Another constraint is the height of the microchannels (h = 35 μm in our design), as the microfluidic device must allow stable and flat embryo positioning inside the incubators, while still avoiding squeezing of the captured embryos. The exact geometry of the embryo incubator plays here an important role, as discussed in detail in Supplementary Note 3. From our experimental analysis, we concluded that the Q_1/Q_2 ratio of ~13.5 guarantees 100% embryo trapping efficiency, while for a ratio of ~4, only 50% of the flowing embryos are typically trapped. We mainly attribute this behavior to the fact that the bypass channel width has to be designed large enough with respect to the embryo size for the reasons explained above.

Another option to reduce mechanical stresses on captured embryos could have been the reduction of the height of the bypass channel with respect to the incubator's one. This would result

in enhanced sealing of the incubator, when occupied by an embryo[3]. However, while spherical objects simply need to be directed towards an empty incubator to be trapped, egg-shaped objects must be properly oriented, in order to ensure their correct capture and positioning in the incubator. By setting equal trap and bypass channel heights, we therefore deliberately allowed the presence of a residual flow through occupied incubators (Figure SI2b). Such residual flow modifies the flow pattern in the vicinity of the subsequent downstream trap and generates an additional flow that is beneficial for guiding the embryo towards its correct re-orientation and trapping into the adjacent empty incubator (Figure SI2c).

Another hydrodynamic trapping design has been recently proposed, in which multiple incubators were positioned side by side in between adjacent serpentine branches[4]. On the one hand, this design undoubtedly allows optimizing the number of captured embryos in a given field of view, but, on the other hand, it does not allow deterministically setting a 100% trapping efficiency, mainly because of the undesired residual flow through occupied incubators, perturbing the expected flow rate through the unoccupied ones. As a result, we considered this alternative design to be less suitable for our application, where the number of available embryos is limited and where trapping efficiency and accuracy are more important than having a larger number of trapping sites.

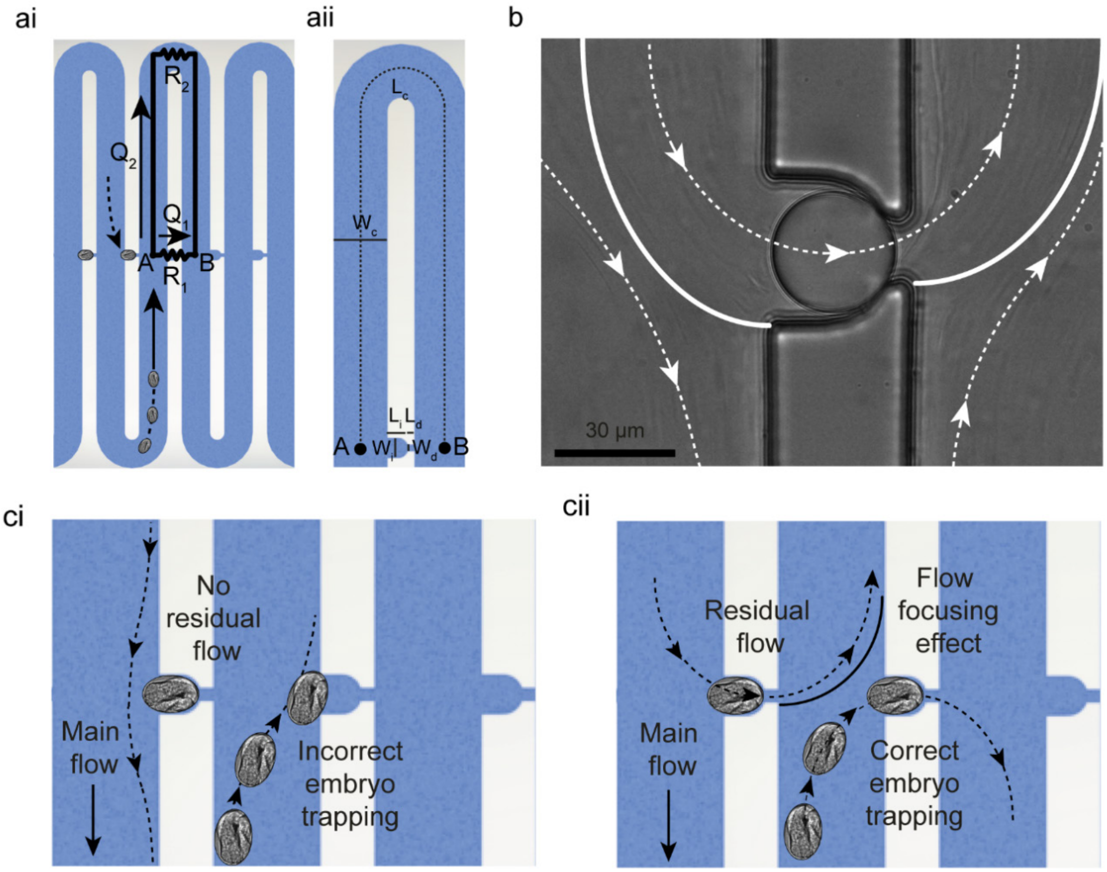

Figure SI2 | Optimization of the hydrodynamic trapping design. (a) Schematic representation of the design of the hydrodynamic embryo trapping system, with (ai) indication of the flow rates Q_1 and Q_2 through a trap and a bypass channel, respectively, and (aii) definition of the geometrical parameters. (b) Optical micrograph of an *E.coli* suspension flowing through an embryo incubator, in which a 30 µm PMMA bead has been trapped to study the hydrodynamic trapping mechanism. Flowing *E.coli* bacteria allow visualizing the flow streamlines and assessing the presence of residual flow through the occupied incubator. Dashed white lines trace a few of the streamlines, while the solid lines are guides to the eye that indicate the boundary between the main flow in the bypass channel and the residual flow in the incubator. (c) Schematic representation of the embryo trapping mechanism, (ci) in absence and (cii) in presence of residual flow through an occupied incubator. The residual flow enhances the correct embryo orientation and positioning inside the next incubator of the array.

Supplementary Note 3. Optimization of the embryo incubator geometry.

The design of an embryo incubator that perfectly matches the requirements of our application is based on the following considerations. The incubator width (W_i in Figure SI2aii and Figure SI3a) has to be large enough to gently accommodate a single *C.elegans* embryo, while not introducing any lateral pressure on it during its complete development. On the other hand W_i cannot be too large with respect to the average width of an embryo, to guarantee its alignment exactly along the same direction, which represents an important requirement for the automated parallel imaging over the whole incubator array. The incubator length (L_i in Figure SI2aii and Figure SI3a) has been chosen taking into account that long incubators would result in multiple embryo trapping at a single incubator site, while too short ones would expose each captured embryo to flow patterns that would change its position during the imaging process or even remove it from the incubator. Dimensions of the drain connecting each incubator to the adjacent branch of the main serpentine channel (W_d and L_d in Figure SI2aii and Figure SI3a) also need to be carefully designed, as *C.elegans* embryos have a limited mechanical stiffness and can be squeezed through the drain by excessive fluidic pressures (Supplementary Video 11). On the one hand, the drain has to be narrow and long enough to retain the embryo inside the incubator, even at the relatively high flow rates used for their capture, while, on the other hand, its fluidic resistance has to be sufficiently low for efficient hydrodynamic trapping. In order to take into consideration all the aforementioned constraints, we designed a dedicated microfluidic device for studying the optimization of the embryo incubator size and shape (Figure SI3b). Our device features parallel arrays of embryo incubators with different geometries for experimentally studying the embryo capture, positioning and imaging for the different designs. *C.elegans* embryo suspensions in M9 buffer were prepared following standard biological protocols[5] and directly injected into the microfluidic device. The various aspects and artefacts, which were theoretically predicted and described above, were experimentally observed indeed (Figure SI3c). Among the tested incubators, we eventually

selected, as optimized design, an elongated semicircular incubator with W_i = 35 µm, L_i = 30 µm, W_d = 12 µm, L_d = 8 µm (Figure 1d, main text). Interestingly, our final design allows moreover emptying the full array in a few seconds, by expelling the captured embryos from the incubators by means of a flow pulse at relatively high flow rate (5-10 µL/s) (Supplementary Video 12).

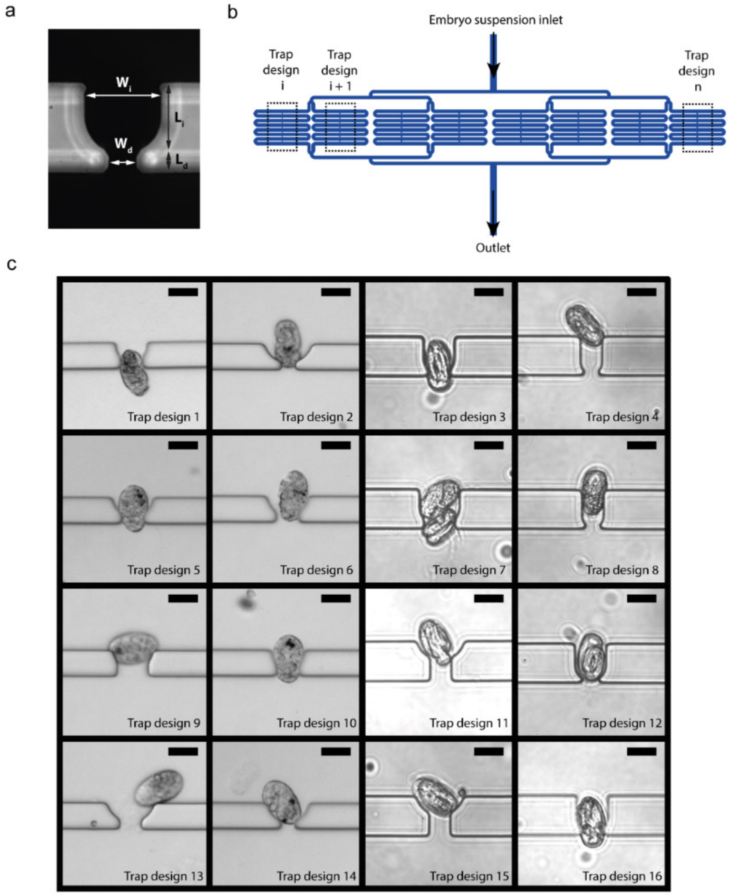

Figure SI3. | Optimization of the embryo incubator geometry. (a) Picture of a PDMS embryo incubator, with main geometric features the incubator width (W_i) and length (L_i), and drain width (W_d) and length (L_d). (b) Schematic representation of the microfluidic device designed and fabricated for the experimental characterization of different embryo incubator geometries. (c) Representative pictures of 16 different embryo incubator geometries, showing possible issues in single embryo trapping, positioning and imaging, such as mechanical stress induced on the captured embryo, wrong embryo positioning/orientation, multiple embryo capture, etc.. Scale bars = 30 µm.

Supplementary Note 4. Analysis of embryo positioning inside the incubators.

Computer-controlled image processing was used on our platform to further extend its analytical possibilities towards fully automated real-time screening and phenotyping. To enable this possibility, however, the exact positioning of each embryo inside the incubator array has to be carefully considered, since image processing tools should always be able to automatically recognize and analyze the embryos in the array. In our device, only slight variations in the embryo angular position inside its incubator are observed (Figure SI4). This variations are dictated by the natural size variability of the embryos themselves, sometimes resulting in slight vertical tilting (Figure SI4, panel E3, where the embryo seems to have a more circular shape resulting from the vertical tilt angle) or horizontal tilting (Figure SI4, panel E9), but always these angles are lower than 10°.

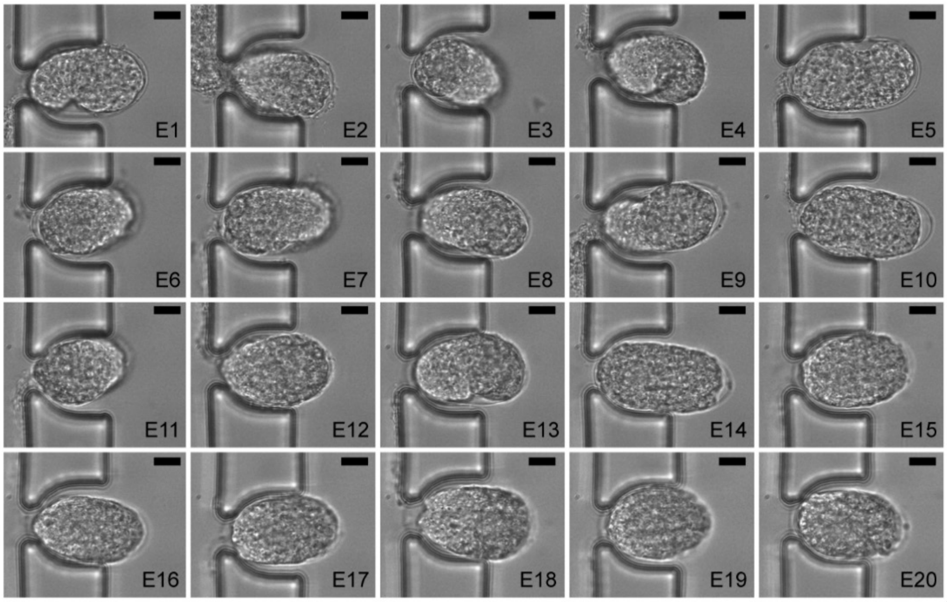

Figure SI4 | Angular position of embryos inside the incubators. Pictures of a representative array of embryos (E1 to E20), upon complete filling of the incubator array. The hydrodynamic trapping mechanism results in stable embryo positioning, with angular deviations within a 5-10° solid angle, due to the natural size and shape variability of the embryos themselves. Scale bars = 10 μm.

Supplementary Note 5. Age-related changes in worm reproduction and progeny.

We demonstrated the capability of our device to be employed for studying age-related changes in worm reproduction and progeny development. Wild-type worms were used for these proof-of-concept experiments. A mixture of L1-L4 larvae was injected into the microfluidic device, to isolate a small population of 5 to 10 L4 larvae inside the worm culture chamber. Worms were then cultured on chip during their full adult lifespan and different embryo populations were isolated at different moments of the worms' reproductive period. As an example, we compared an array of embryos shortly captured after the start of egg production (Day 1, Figure 3di of main article) with another array that has been filled approximately 72 hours later (Day 4, Figure SI5). For the first generation of embryos (Day 1), about 50% of the captured eggs were in phase 1 or phase 2 (i.e. before the bean stage or before the onset of twitching, respectively). As worms get older, however, we observed a general decrease in egg production and brood viability, as well as an increase of the average age of the embryos at laying. These observations are in good agreement with off-chip results (data not shown).

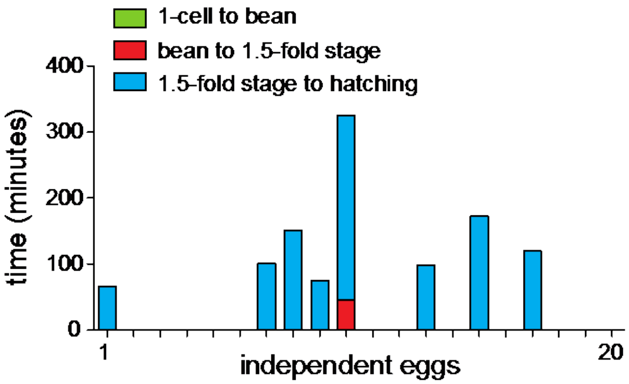

Figure SI5 | Age-related changes in worm reproduction and progeny development. Duration of development phases 72 hours after the start of egg production, as observed for an array of 20 embryos for a N2 wild-type worm strain at 25°C. As worms get older, a general decrease in egg production and brood viability is observed, as well as an increase of the average age of the embryos at laying. Absence of a bar in the histogram indicates a non-viable embryo.

Supplementary note 6. Duration of the different development phases for transgenic strains and mutants.

In Fig. 3e of the main text, we report a systematic study of the duration of the different development phases for several transgenic strains and mutants. In particular, we used SJ4100, SJ4103 and SJ4143 transgenic strains of worms, which express Green Fluorescent Protein (GFP) under the control of the *hsp-6* (homolog of mtHSP70 in mouse), the *myo-3* (homolog of Myosin-3 in mouse) and the *ges-1* (homolog of Carboxylesterase in mouse) promoters, respectively, in a N2 wild-type background[6, 7]. These strains are typically employed for the study of the gene expression level and/or localization, either through the GFP quantification or imaging approaches. Also we used the transgenic strain AGD1073 obtained by crossing *hsp-6::GFP* reporter worms with a neuron-specific *cco-1* hairpin line[8]. The resulting *Prab-3::cco-1HP;hsp-6::gfp* strain of worms displays a specific *cco-1* (homolog of the cytochrome c oxidase-1 subunit Vb/COX4 in mouse) silencing in the neurons driven by the pan-neuronal promoter *rab-3*. Finally, we investigated the TK22, DA465 and MQ887 strains, carrying a loss-of-function mutation in the *mev-1, eat-2* and *isp-1* genes, respectively[9-11]. *mev-1* encodes the cytochrome *b* large subunit (Cyt-1/ceSDHC) in complex II of the mitochondrial electron transport chain. The *mev-1* mutation was shown to shorten lifespan and the mutants display a large panel of age-related phenotypes and metabolic disorders[12]. *eat-2* encodes a ligand-gated ion channel subunit closely related to the non-alpha-subunits of nicotinic acetylcholine receptors (nAChR). Mutation in the *eat-2* gene affects primarily pharyngeal pumping rates, slowing pumping down to 10–20% of the normal rhythm, which mimics a caloric restriction state and increases the lifespan[13]. *isp-1* encodes a Rieske iron sulphur protein, which is a subunit of complex III of the mitochondrial electron transport chain. A mutation in this gene affects the rates of physiological processes like reproduction and development, and extends the longevity[11].

For the *mev-1* mutants, we observed that egg laying was delayed, so that these embryos could be trapped only after reaching the bean stage. Therefore, phase 2 could not be completely monitored for all embryos, as they were not captured right from the beginning, and the duration of phase 2, as indicated in Fig. 3e, corresponds to that of the embryo for which phase 2 could be observed longest. However, phase 3 could be accurately determined for all *mev-1* mutant embryos and was significantly prolonged with respect to wild-type organisms (360 min).

Supplementary Note 7. Mitochondrial stress in the *isp-1(qm150);hsp-6::gfp* transgenic strain at the adult stage.

Studies performed at the larval stages indicated that the UPR^{mt} can be robustly induced by applying direct mitochondrial stress[8]. These stresses include oxidative stress and the inhibition of the mitochondrial transcription and translation machinery, resulting in the disturbance of the mitonuclear proteostatic balance[6,14,15]. Mitonuclear imbalance results from the disruption of the stoichiometric balance between components of oxidative phosphorylation (OXPHOS) complexes encoded by the mitochondrial and/or the nuclear genome and robustly activates the UPR^{mt} [15]. Examples of such a mitonuclear imbalance are provided by the loss-of-function mutation of the nuclear-encoded complex III protein, *isp-1,* and/or the complex IV protein, *cco-1*, which induces a robust UPR^{mt} and extends worm lifespan. These genetic manipulations deplete single components of specific OXPHOS complexes, overloading the mitochondrial matrix with their respective partner proteins, which cannot be assembled into multiprotein complexes.

We generated a transgenic strain carrying the *isp-1(qm150)* mutation and the *hsp-6::gfp* reporter, by crossing the SJ4100 (*hsp-6::gfp*) and MQ887 (*ips-1(qm150)*) strains. The resulting transgenic worms showed a constitutive activation of *hsp-6::gfp*, compared to the unstressed *hsp-6::gfp* control population after one day of adulthood (Fig. SI6).

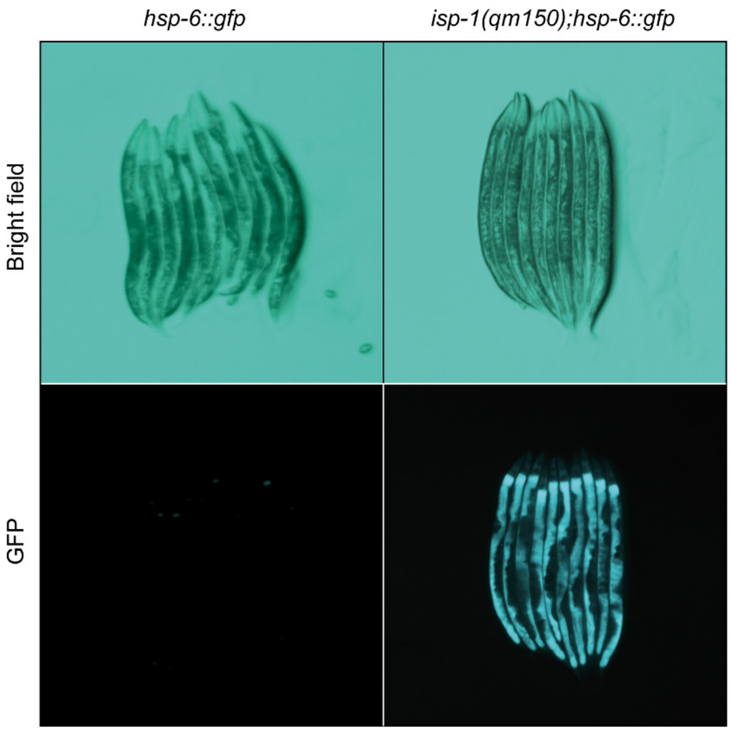

Figure SI6 | A mutation in the mitochondrial gene *isp-1* induces a robust UPR^{mt} in the post-embryonic life. Pictures of the transgenic strain of worms *isp-1(qm150);hsp-6::gfp* at day 1 of the adulthood, showing a UPR^{mt} induction, as revealed by the robust GFP expression, and comparison with the control *hsp-6::gfp*.

Supplementary Note 8. Mitochondrial stress in the *Prab-3::cco-1HP;hsp-6::gfp* transgenic strain.

In this strain, the down-regulation of *cco-1* in neuronal cells induces not only a localized mitochondrial stress and UPR^{mt} in the nervous system, but also induces a so-called “mitokine” signal, which will relay and induce UPR^{mt} in peripheral tissues, such as the intestine[8]. This mitochondrial communication between central and distal tissues seems to be fundamental, as it can synchronize the rate of aging for the whole organism independently of the cell-autonomous functions[16]. In this regard, we asked whether a similar mitochondrial cross-talk between tissues would already occur during the embryogenesis. One would in fact expect that the perfect organisation of this developmental period could involve a watchful communication between tissues in terms of mitochondrial biogenesis and stress response regulation.

References

1. Tan, W.H. & Takeuchi, S. A trap-and-release integrated microfluidic system for dynamic microarray applications. *P Natl Acad Sci USA* **104**, 1146-1151 (2007).
2. Bruus, H. Theoretical Microfluidics. (Oxford Univ Press, New York; 2008).
3. Kobel, S., Valero, A., Latt, J., Renaud, P. & Lutolf, M. Optimization of microfluidic single cell trapping for long-term on-chip culture. *Lab on a chip* **10**, 857-863 (2010).
4. Chung, K. et al. A microfluidic array for large-scale ordering and orientation of embryos. *Nature Methods* **8**, 171-U103 (2011).
5. Porta-de-la-Riva, M., Fontrodona, L., Villanueva, A. & Ceron, J. Basic Caenorhabditis elegans methods: synchronization and observation. *Journal of visualized experiments : JoVE*, e4019 (2012).
6. Yoneda, T. et al. Compartment-specific perturbation of protein handling activates genes encoding mitochondrial chaperones. *Journal of cell science* **117**, 4055-4066 (2004).
7. Benedetti, C., Haynes, C.M., Yang, Y., Harding, H.P. & Ron, D. Ubiquitin-like protein 5 positively regulates chaperone gene expression in the mitochondrial unfolded protein response. *Genetics* **174**, 229-239 (2006).
8. Durieux, J., Wolff, S. & Dillin, A. The cell-non-autonomous nature of electron transport chain-mediated longevity. *Cell* **144**, 79-91 (2011).
9. Ishii, N. et al. A methyl viologen-sensitive mutant of the nematode Caenorhabditis elegans. *Mutation research* **237**, 165-171 (1990).
10. Raizen, D.M., Lee, R.Y. & Avery, L. Interacting genes required for pharyngeal excitation by motor neuron MC in Caenorhabditis elegans. *Genetics* **141**, 1365-1382 (1995).
11. Feng, J., Bussiere, F. & Hekimi, S. Mitochondrial electron transport is a key determinant of life span in Caenorhabditis elegans. *Developmental cell* **1**, 633-644 (2001).

12. Ishii, N. et al. A mutation in succinate dehydrogenase cytochrome b causes oxidative stress and ageing in nematodes. *Nature* **394**, 694-697 (1998).

13. Lakowski, B. & Hekimi, S. The genetics of caloric restriction in Caenorhabditis elegans. *Proc Natl Acad Sci USA* **95**, 13091-13096 (1998).

14. Runkel, E.D., Liu, S., Baumeister, R. & Schulze, E. Surveillance-activated defenses block the ROS-induced mitochondrial unfolded protein response. *PLoS genetics* **9**, e1003346 (2013).

15. Houtkooper, R.H. et al. Mitonuclear protein imbalance as a conserved longevity mechanism. *Nature* **497**, 451-457 (2013).

16. Taylor, R.C., Berendzen, K.M. & Dillin, A. Systemic stress signalling: understanding the cell non-autonomous control of proteostasis. *Nature reviews. Molecular cell biology* **15**, 211-217 (2014).
